# Natural disease history of the *dy^2J^* mouse model of laminin α2 (merosin)-deficient congenital muscular dystrophy

**DOI:** 10.1371/journal.pone.0197388

**Published:** 2018-05-15

**Authors:** S. Pasteuning-Vuhman, K. Putker, C. L. Tanganyika-de Winter, J. W. Boertje-van der Meulen, L. van Vliet, M. Overzier, J. J. Plomp, A. Aartsma-Rus, M. van Putten

**Affiliations:** 1 Department of Human Genetics Leiden University Medical Centre, Leiden, The Netherlands; 2 Department of Neurology Leiden University Medical Centre, Leiden, The Netherlands; University of Minnesota Medical Center, UNITED STATES

## Abstract

Merosin deficient congenital muscular dystrophy 1A (MDC1A) is a very rare autosomal recessive disorder caused by mutations in the *LAMA2* gene leading to severe and progressive muscle weakness and atrophy. Although over 350 causative mutations have been identified for MDC1A, no treatment is yet available. There are many therapeutic approaches in development, but the lack of natural history data of the mouse model and standardized outcome measures makes it difficult to transit these pre-clinical findings to clinical trials. Therefore, in the present study, we collected natural history data and assessed pre-clinical outcome measures for the *dy*^*2J*^*/dy*^*2J*^ mouse model using standardized operating procedures available from the TREAT-NMD Alliance. Wild type and *dy*^*2J*^*/dy*^*2J*^ mice were subjected to five different functional tests from the age of four to 32 weeks. Non-tested control groups were taken along to assess whether the functional test regime interfered with muscle pathology. Respiratory function, body weights and creatine kinase levels were recorded. Lastly, skeletal muscles were collected for further histopathological and gene expression analyses. Muscle function of *dy*^*2J*^*/dy*^*2J*^ mice was severely impaired at four weeks of age and all mice lost the ability to use their hind limbs. Moreover, respiratory function was altered in *dy*^*2J*^*/dy*^*2J*^ mice. Interestingly, the respiration rate was decreased and declined with age, whereas the respiration amplitude was increased in *dy*^*2J*^*/dy*^*2J*^ mice when compared to wild type mice. Creatine kinase levels were comparable to wild type mice. Muscle histopathology and gene expression analysis revealed that there was a specific regional distribution pattern of muscle damage in *dy*^*2J*^*/dy*^*2J*^ mice. Gastrocnemius appeared to be the most severely affected muscle with a high proportion of atrophic fibers, increased fibrosis and inflammation. By contrast, triceps was affected moderately and diaphragm only mildly. Our study presents a complete natural history dataset which can be used in setting up standardized studies in *dy*^*2J*^*/dy*^*2J*^ mice.

## Introduction

Congenital muscular dystrophy (CMD) is a very rare and highly heterogeneous group of autosomal recessive disorders that can be subdivided into eight genetically distinct forms [1]. Laminin-α2 chain (merosin) deficient congenital muscular dystrophy 1A (MDC1A) is the most prevalent form of CMD, caused by mutations in the *LAMA2* gene [2]. Over 350 different mutations in the *LAMA2* gene have been identified causing laminin-α2 chain deficiency (www.dmd.nl) [3]. Generally, complete absence of the laminin-α2 chain leads to a very severe disease course, while partial deficiency results in a milder phenotype [3, 4]. Due to complete or partial absence of the laminin-α2 chain, skeletal muscles of MDC1A patients display typical dystrophic features including muscle degeneration and regeneration, inflammation, atrophy and fibrosis [5]. Besides skeletal muscles, also other tissues are affected including the brain, Schwann cells, heart and lungs [5]. Several mouse models for MDC1A have been developed, which recapitulate many characteristics of the disease and confirm correlation between laminin-α2 chain expression and disease severity [5–8]. Complete absence of laminin-α2 chain in *dy*^*W*^*/dy*^*W*^ and *dy* ^*3K*^*/dy* ^*3K*^ mice leads to a very severe muscular dystrophy, peripheral neuropathy and reduced lifespan. Partial reduction of the laminin-α2 chain in *dy*^*2J*^*/dy*^*2J*^ mice, on the other hand, leads to mild muscular dystrophy with severe peripheral neuropathy and a less reduced lifespan [9]. Analyses of these mouse models provided better understanding about disease driving mechanisms. However, with a therapy lacking, there is a growing demand for the development of therapies. Although many therapeutic approaches have been tested in mouse models, it is difficult to compare the studies due to the use of different outcome measures [5, 9]. To facilitate and improve drug development in MDC1A mouse models, generally accepted pre-clinical outcome measures and natural history data for these outcome measures are required. Global recognition in the field catalysed an international effort initiated by the TREAT-NMD Alliance and the Wellstone Center to develop standardized procedures for the *dy*^*W*^*/dy*^*W*^ model for CMD (http://www.treat-nmd.eu/research/preclinical/cmd-sops/). These standardized procedures can be used worldwide allowing more direct comparison of pre-clinical findings between laboratories (http://www.treat-nmd.eu/research/preclinical/overview/). However, these measures are of limited use for the less severely affected models with partial laminin-α2 chain deficiency like the *dy*^*2J*^*/dy*^*2J*^ mouse model. For these, there is a shortage of consensus on accepted pre-clinical outcome measures and consequently a lack of natural history data. To address this, we characterized the natural disease history of *dy*^*2J*^*/dy*^*2J*^ mice using standardized operating procedures available from the TREAT-NMD Alliance for the *mdx* mouse of Duchenne muscular dystrophy. These data can be suitable for setting up standardized pre-clinical studies in *dy*^*2J*^*/dy*^*2J*^ mice.

## Materials and methods

### Animals

The *dy*^*2J*^*/dy*^*2J*^ (B6.WK-*Lama2*^*dy-2J*^/J) mice were purchased from Jackson Laboratory (strain 000524) (10) and bred in the Experimental Animal Facility of the Leiden University Medical Center. The mice were received on a C57BL/6J background and could therefore be compared to C57BL/6J wild type mice. They were kept in individually ventilated cages with 12 hours of light/dark cycles at 20.5°C and had *ad libitum* access to standard RM3 chow (SDS, Essex, UK) and water. Animal Experiment Committee (Dierexperimentencommissie) of the Leiden University Medical Center approved all performed experiments. Efforts were made to minimize the burden and distress.

### Functional test regime

**Fig 1:**
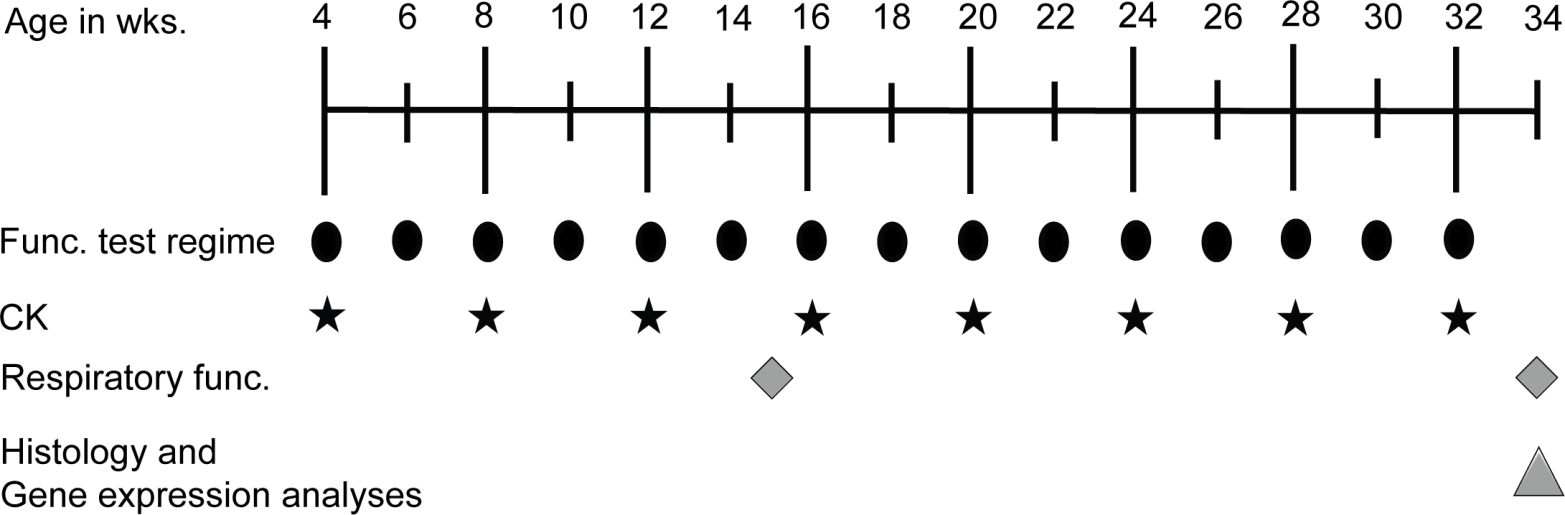
Experimental timeline. Mice were subjected to the functional test regime every other week. Time points are indicated by the black ellipses. Black stars donate time points at which creatine kinase (CK) levels were measured. Grey rhombuses indicate time points at which the respiratory function was assessed. The grey triangle denotes time point at which mice were sacrificed and terminal analyses were performed.

The *dy*^*2J*^*/dy*^*2J*^ mice were randomized and assigned to a functionally challenged and sedentary group (12 mice per group; six males and six females). As the functional test regime is not detrimental to C57BL/6J mice [10], these were assigned to the functionally challenged group only (six males and six females). Functionally challenged mice were subjected to a test regime of five functional tests performed on consecutive days in the afternoon twice monthly from the age of four to 32 weeks, as previously described [11]. These tests assessed muscle function, strength, coordination and condition. Before each functional test regime session, body weight was recorded. At 34 weeks of age, mice were sacrificed by cervical dislocation and muscles were isolated and snap frozen in 2-methylbutane (Sigma-Aldrich), cooled in liquid nitrogen and stored at -80°C until further processing. A schematic overview of the experimental timeline is presented in Fig 1. Standardized operating procedures from the TREAT-NMD Alliance available for *mdx* mice were applied when possible [12].

#### Four limb grip strength test

Peak force of the four limbs was measured using a grid attached to an isometric force transducer (Columbus Instruments, USA). Hereto, the mouse was suspended, handled by the tail, above the grid which it naturally grasped with four paws. The mouse was then pulled away from the grid and the force transducer recorded the maximal force applied by the mouse to the grid [11]. The measurement was performed three times in a row after which the mouse received a short break. In total each mouse was subjected to five series of three pulls, each followed by a resting period of two minutes. In total each mouse pulled 15 times. The three highest out of these 15 values were averaged and this maximum grip strength was normalized to body weight.

#### Rotarod

Rotarod running assesses motor coordination and balance. The mouse was put on the rotarod (Ugo Basile, Italy) that accelerated from 5 to 45 rotations per minute (rpm) within 15 seconds. The time it took mice to fall off the rod was measured. The test was completed after a running time of 500 seconds was achieved [11]. If a mouse did not reach 500 seconds, it was allowed two more tries. The longest running time was used for analysis.

#### Two limb hanging test

The mouse was suspended above a metal wire which was secured ~40 cm above a cage with bedding and released after it had grasped the wire with its fore limbs. Mice were allowed to hold onto the wire with all four limbs and the tail, if capable to do so. Directly after a mouse dropped from the wire, recording of the hanging time was stopped. The test was completed after a hanging time of 600 seconds was achieved or after three sessions [11]. The maximum time was used for analysis.

#### Four limb hanging test

The mouse was placed on a grid, which was then turned upside down 15 cm above a cage filled with bedding. The test was completed after a hanging time of 600 seconds was achieved or after three sessions [11]. The maximum hanging time was used for analysis.

#### Beam walk test

The mouse was put on an elevated narrow beam (diameter 11mm) and had to cross the beam to be able to reach a safe black box, while being filmed. Performance score was calculated by the time it took to traverse the 80 cm long beam. The numbers of hind paw slips that took place during beam walking was quantified [11]. The average of the traverse times and numbers of paw slips from three tries were calculated.

#### Respiratory function analysis

Respiratory function was assessed with whole-body plethysmography (RM-80; Columbus Instruments, Columbus, OH, USA) [13] at the age of 15 and 34 weeks. After 30 seconds acclimatization, the respiration signal was measured for 120 seconds. The signal was loaded using a MiniDigi digitizer and AXOSCOPE 10 software (Axon Instruments/Molecular Devices, Sunnyvale, CA, USA) and analysed with the event detection feature of the Clampfit 10 program (Axon Instruments/Molecular Devices). Using this non-invasive monitoring system, respiration rate and amplitude were recorded. The respiration amplitude was normalized to body weight [11]. Respiratory function data of n = 5 male and n = 2 female wild type mice aged 34 weeks was excluded due to a broken seal of the plethysmography apparatus. Consequently, we have collected respiratory function data of age, gender and genotype matched wild type mice to make up for this. For the other n = 1 male and n = 4 female wild type mice longitudinal data is provided. Data collection of the *dy*^*2J*^*/dy*^*2J*^ mice was not affected by this.

### Creatine kinase level analysis

Once monthly, a few hours prior to the first functional test, blood was collected via a small angled cut in the tail in a Heparin coated Microvettes CB 300 (Sarstedt B.V. the Netherlands) and stored on ice. Samples were centrifuged at 4°C for five minutes at 13000 rpm and the obtained plasma was used to measure creatine kinase (CK) levels with Reflotron CK test strips in the Reflotron plus machine (Roche Diagnostics Ltd., UK).

### Muscle histology and morphology

Using a cryotome, frozen muscle sections (8 μm thick) were cut for histological analysis and intermediate sections were collected for RNA isolation. For analyses of pathological tissue, slides were fixed in ice-cold acetone for five minutes, stained with haematoxylin and eosin (Sigma-Aldrich, Zwijndrecht, the Netherlands) and mounted in Pertex mounting medium (Histolab, Västra Frölunda, Sweden) [11]. Images of the stained muscle sections were taken with a BZ-X700 fluorescent microscope (Keyence, Osaka, Japan) at ten times magnification. The images were stitched using BZ-X Analyzer (Keyence). Using ImageJ, the pathological areas (inflammation, degeneration, regeneration and fibrosis) were measured as previously described [10]. To quantify the levels of collagen, the muscle sections were fixed in 4% paraformaldehyde for ten minutes, and in 100% ethanol for five minutes and air dried for 30 minutes. After being rinsed in deionized water, slides were stained with Sirius Red solution (Sigma-Aldrich) for 45 minutes. After this, the stained sections were washed with 0.5% acetic acid water followed by rinsing with deionized water. The stained sections underwent dehydration steps and were mounted in Pertex mounting medium (Histolab). The stained muscle sections were imaged using a BZ-X700 fluorescent microscope (Keyence) at ten times magnification and stitched using BZ-X Analyzer (Keyence). The Sirius red positive areas were quantified using ImageJ and normalized to the total area. To quantify number and size of fibers, muscle sections were stained with a laminin primary antibody (ab11575, dilution 1:100 Abcam, USA) and with a goat-anti-rabbit Alexa 594 secondary antibody (A11012, dilution 1:1000, Life Technologies, Bleiswijk, the Netherlands). Five pictures taken at a 10 times magnification were analysed with BZ-X Analyzer (Keyence) resulting in an average total number of 1700–3500 and 1000–3000 fibers per muscle for wild type and *dy*^*2J*^*/dy*^*2J*^ mice, respectively, measured of which the number of fibers in a given fiber area class (500 μm^2^/class) was determined.

### RNA isolation and qPCR

Muscle sections were collected in 1.4mm Zirconium Beads prefilled tubes (OPS Diagnostics, Lebanon, USA) and were homogenized in TRIsure isolation reagent (Bioline, London, United Kingdom) using a MagNA Lyser (Roche Diagnostics). Total RNA was isolated using the TRIsure isolation method. The RNA was further purified (including DNase digestion step) by applying a NucleoSpin RNA II kit (Macherey-Nagel, Düren, Germany) according to the manufacturer's instruction. From 0.4μg of RNA cDNA was synthesized using random N6 primers (Thermo fisher scientific) and Bioscript enzyme (GCBiotech, Alphen aan den Rijn, the Netherlands) according to the manufacturer's instructions. Quantitative PCR was conducted in triplo per biological sample with the use of LightCycler 480 and the ready to use SensiMix reagents (GCBiotech). The expression levels were analysed applying the LinReg qPCR method [14] and normalized to the housekeeping gene *Gapdh*. Primer sequences can be found in S1 Table.

### Statistical analyses

Data were analysed using Prism 4 (GraphPad Software, La Jolla, CA, USA) and SPSS 17.0.2 (IBM, Armonk, NY, USA). Values are presented as mean ± SD (standard deviation) or ± SEM (standard error of the mean), as indicated. To compare limb grip strength, maximum hanging time from two and four limb hanging tests and maximum running time from rotarod, CK levels and body weights over time per mouse per genotype, a linear regression model was applied. To compare respiration rate and amplitude between genotypes, the one-way ANOVA test was utilized and corrected for multiple comparisons with Tukey’s test. Measurements from histological and gene expression analyses were compared between wild type and *dy*^*2J*^*/dy*^*2J*^ mice using the one-way ANOVA test and corrected for multiple comparisons with Tukey’s test. To assess fiber size distribution, a logistic regression was conducted with SPSS to demonstrate the switch toward smaller fiber sizes for *dy*^*2J*^*/dy*^*2J*^ mice. Statistical significance was set at *P* < 0.05.

## Results

### Decreased body weight, impaired muscle function and integrity in merosin-deficient *dy*^*2J*^*/dy*^*2J*^ mice

**Fig 2:**
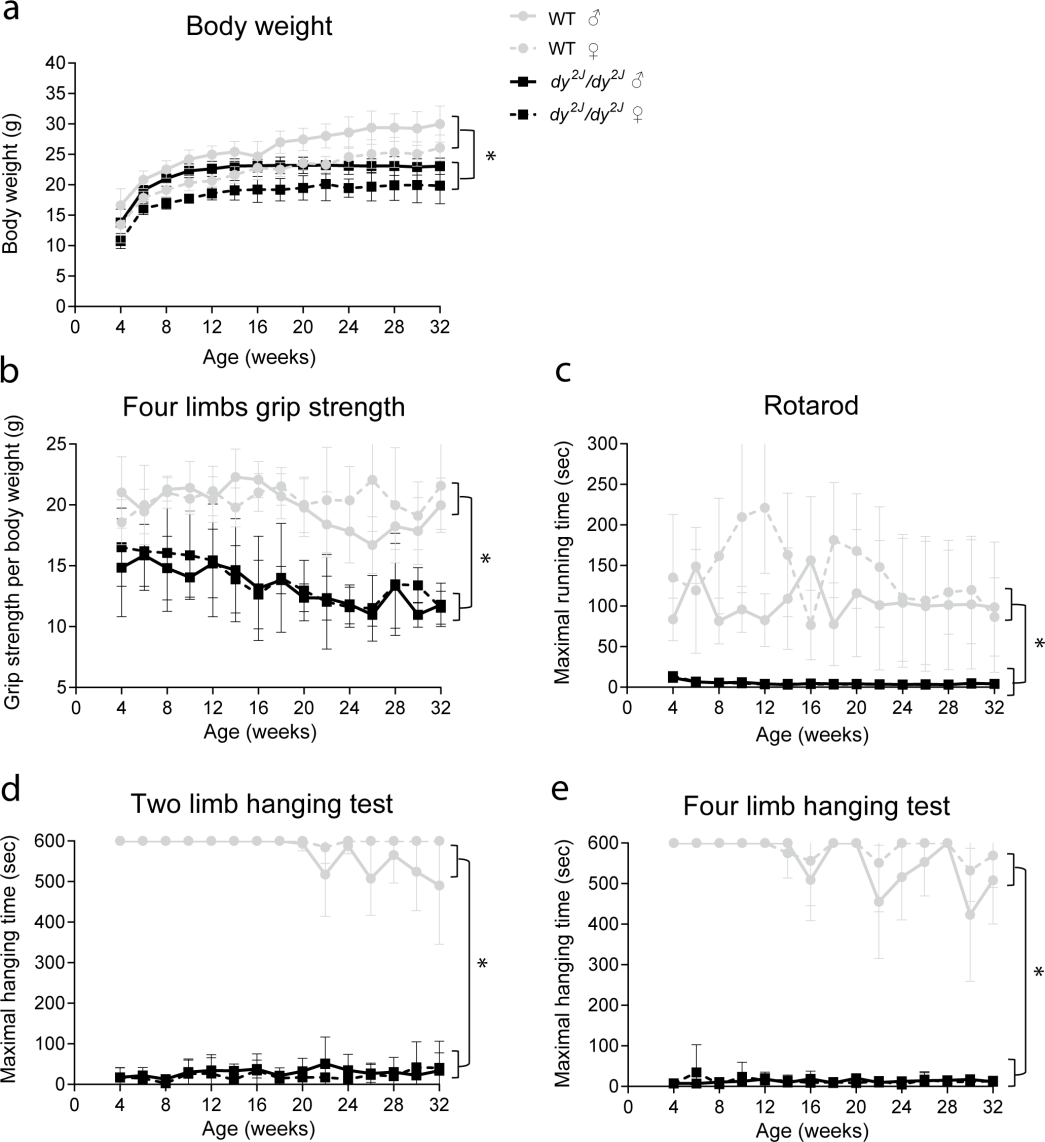
Impaired muscle function in *dy*^*2J*^*/dy*^*2J*^ mice. **(a)** A lower body weight (*P*<0.01) was detected in *dy*^*2J*^*/dy*^*2J*^ mice when compared to wild type mice. **(b)** Normalized four limb grip strength was lower (*P*<0.0001) and decreased with age in *dy*^*2J*^*/dy*^*2J*^ compared to wild type mice. **(c)** *Dy*^*2J*^*/dy*^*2J*^ mice ran for shorter time (*P*<0.001) than wild type mice. **(d)** *Dy*^*2J*^*/dy*^*2J*^ mice hung for shorter time (*P*<0.0001) compared to wild type mice. **(e)** Maximum hanging time with four limbs was shorter (*P*<0.0001) in *dy*^*2J*^*/dy*^*2J*^ mice when compared to wild type mice. * Indicates a significant difference from wild type (WT) controls. Error bars represent ± SEM and n = 6 per genotype per gender.

Twice monthly, wild type and *dy*^*2J*^*/dy*^*2J*^ mice were subjected to a functional test regime from four to 32 weeks of age and body weights were recorded. *Dy*^*2J*^*/dy*^*2J*^ mice were significantly lighter than gender-matched wild type mice (Fig 2A). The functional test regime was composed of: four limb grip strength, rotarod running, two and four limb hanging tests and beam walk test. To assess muscle strength and function, the four limb grip strength test was used revealing significantly lower grip strength in *dy*^*2J*^*/dy*^*2J*^ compared to wild type mice (Fig 2B). In the rotarod test, which examines coordination and endurance, *dy*^*2J*^*/dy*^*2J*^ mice ran for a significantly shorter time period than wild type mice. In fact, most of the *dy*^*2J*^*/dy*^*2J*^ mice were unable to perform this test at all. For wild type mice, this test showed a very high inter-individual variation (Fig 2C). Muscle strength and fatigability were assessed with two and four limb hanging tests. In these hanging tests, *dy*^*2J*^*/dy*^*2J*^ mice hung for a significantly shorter time period than wild type mice (Fig 2D and 2E). Notably, *dy*^*2J*^*/dy*^*2J*^ mice had very low scores in all performed tests from four weeks of age onwards and they were not able to accomplish the beam walk test due to hind limb paralysis. No significant differences in any of these tests were found between genders.

**Fig 3:**
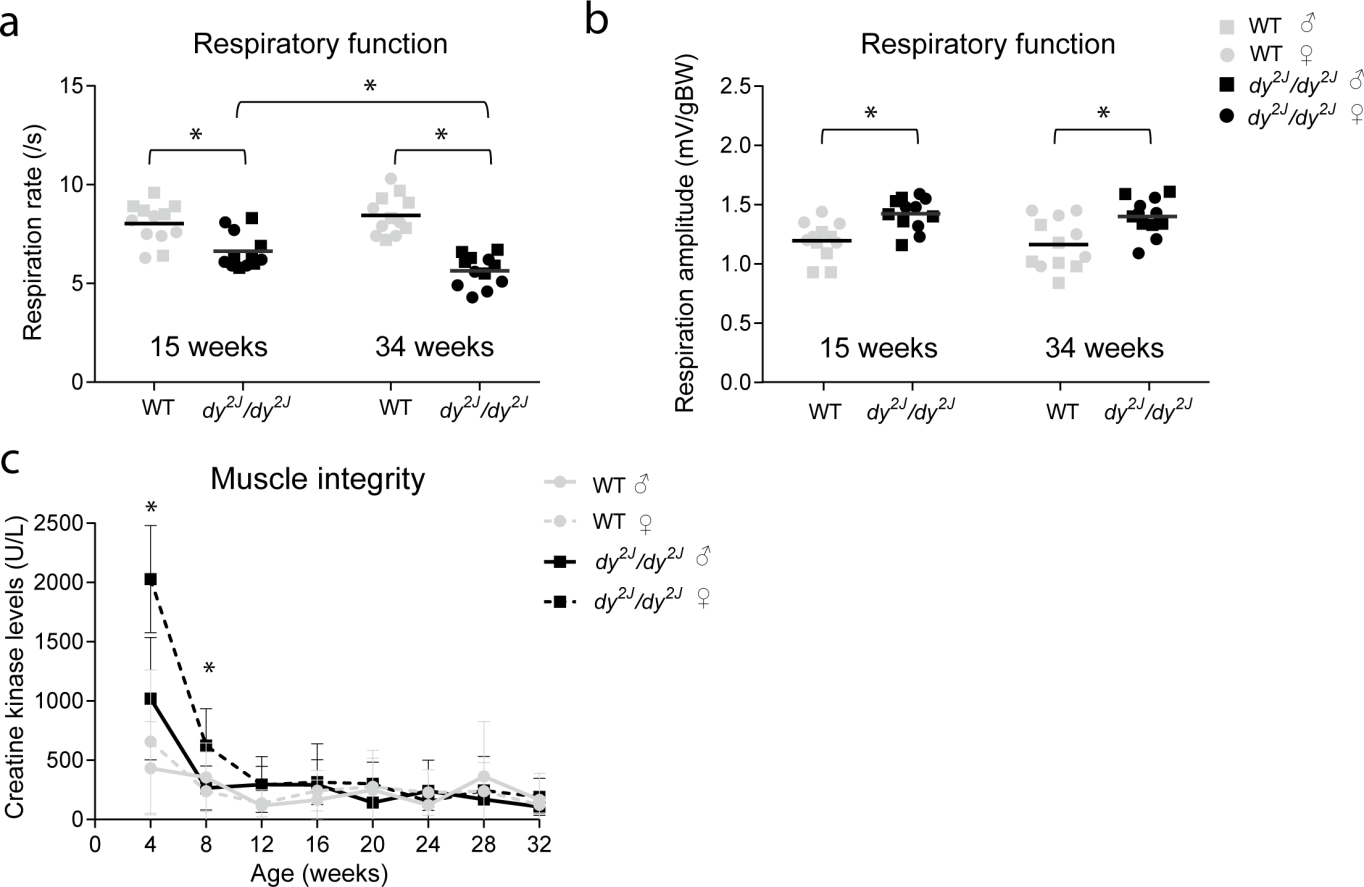
Respiratory function and creatine kinase levels in *dy*^*2J*^*/dy*^*2J*^ mice. **(a)** Respiration rate was significantly lower (*P*<0.001) in *dy*^*2J*^*/dy*^*2J*^ mice at 15 and 34 weeks of age, as compared to wild type mice (WT). *Dy*^*2J*^*/dy*^*2J*^ mice aged 34 weeks showed a significantly lower (*P*<0.05) respiration rate than 15 weeks old mice, while no differences were found with age in wild type mice. **(b)** Respiration amplitude normalized to body weight was significantly higher (*P*<0.001) in *dy*^*2J*^*/dy*^*2J*^ mice at 15 and 34 weeks of age, as compared to wild type mice. No differences in normalized respiration amplitude were found with age and between genders in wild type and *dy*^*2J*^*/dy*^*2J*^ mice. Males are indicated by squares and females by circles. **(c)** Creatine kinase levels were significantly higher (*P*<0.01) in females *dy*^*2J*^*/dy*^*2J*^ and wild type mice at four and eight weeks of age. * Indicates a significant difference. Error bars represent ± SEM, n = 6 per genotype per gender.

To assess whether respiratory function was impaired, mice underwent respiratory function analysis with whole-body plethysmography. The respiration rate was significantly lower in *dy*^*2J*^*/dy*^*2J*^ mice at both ages, compared to wild type mice. The rate further declined with age in *dy*^*2J*^*/dy*^*2J*^ mice, whereas it remained stable in wild type mice (Fig 3A). The body weight-normalized respiration amplitude was significantly increased in *dy*^*2J*^*/dy*^*2J*^ mice, relative to wild type mice at both ages (Fig 3B). In contrast to the rate, respiratory amplitude did not decline with age. No differences in respiration rate or amplitude were found between genders. We longitudinally assessed plasma CK levels of the mice as a marker for muscle fiber integrity. Significant differences were found in CK levels between *dy*^*2J*^*/dy*^*2J*^ females and wild type mice at age of four and eight weeks (Fig 3C). From twelve weeks onwards, however, CK levels were comparable between genotypes.

To summarize, *dy*^*2J*^*/dy*^*2J*^ mice were smaller, performed poorly in five functional tests and their muscle strength deteriorated over time. The mice developed paralysis of the hind limbs from six weeks of age onwards which became more severe with age. The hind limb paralysis interfered with the performance on the beam walk and they were not able to comply with that test. Respiratory function was impaired already from 15 weeks of age onwards and deteriorated over time. Plasma CK levels were elevated at four and eight weeks of age but normalized with age.

### Histological examination and gene expression analyses

#### Muscle atrophy is present in *dy*^*2J*^*/dy*^*2J*^ mice

**Fig 4:**
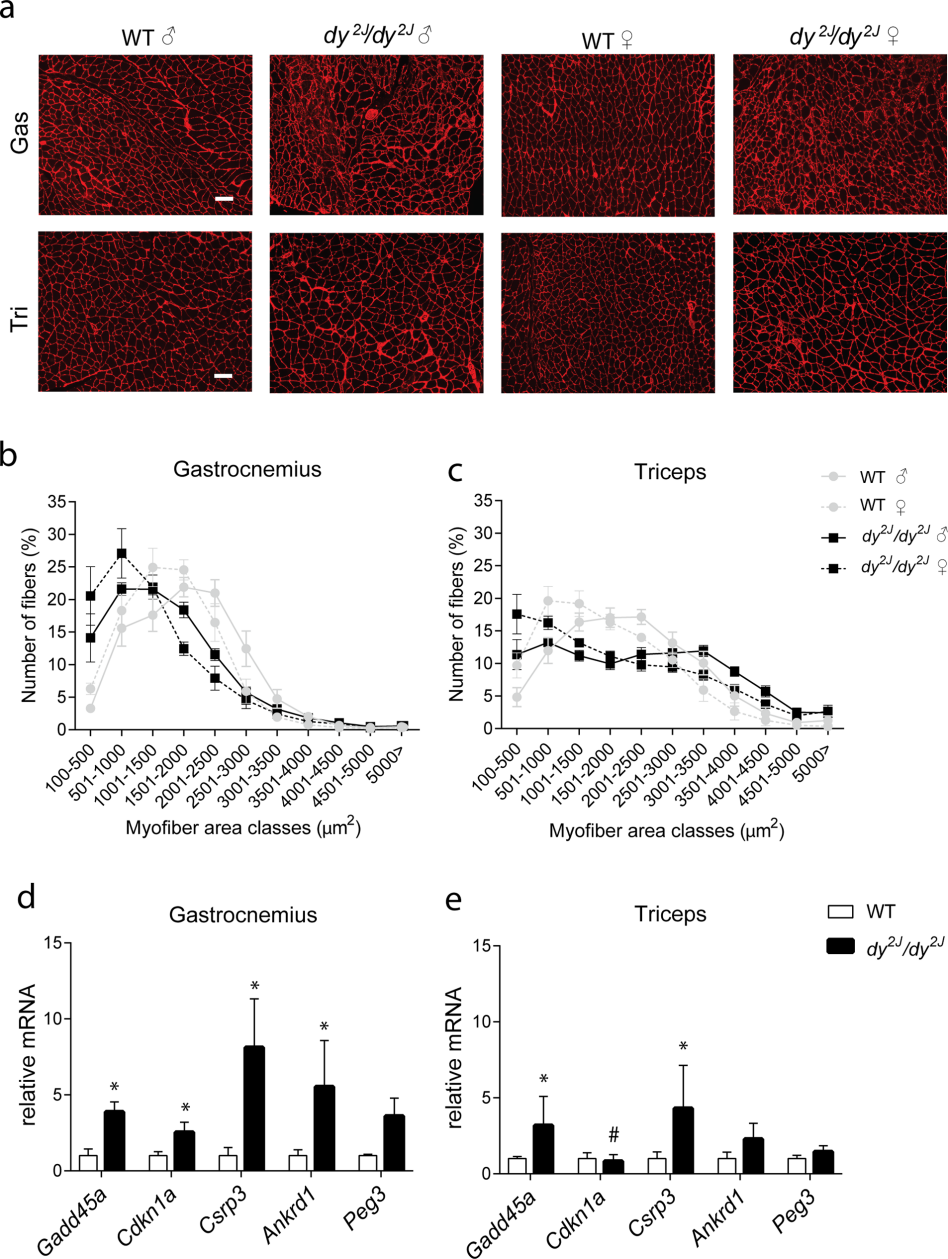
Muscle atrophy in *dy*^*2J*^*/dy*^*2J*^ mice. **(a)** Representative pictures of laminin-stained gastrocnemius and triceps from 34 weeks old male *dy*^*2J*^*/dy*^*2J*^ and wild type mice. Scale bar: 100 μm **(b-c)** Fiber size distribution of skeletal muscles of 34-week old and *dy*^*2J*^*/dy*^*2J*^ and wild type mice. Values represent relative number of fibers in a given area class (500 μm^2^/class). Gastrocnemius and triceps from *dy*^*2J*^*/dy*^*2J*^ mice displayed increased number of smaller fibers when compared to wild type mice. n = 5 mice per genotype per gender **(d)** In *dy*^*2J*^*/dy*^*2J*^ gastrocnemius, *Gadd45a*, *Cdkn1a*, *Csrp3* and *Ankrd1* mRNA levels were significantly increased (*P*<0.01) compared to wild type muscle. **(e)** In *dy*^*2J*^*/dy*^*2J*^ triceps, *Gadd45a* and *Csrp3* mRNA levels were significantly increased (*P*<0.01) compared to wild type muscle. Gene expression of atrophic markers measured by qPCR, normalized to *Gapdh*. Gas, gastrocnemius; Tri, triceps. * Indicates a significant difference from muscle type-matched wild type (WT) controls. # Indicates a significant difference from *dy*^*2J*^*/dy*^*2J*^ gastrocnemius muscle. Error bars represent ±SD, n = 5 (functionally challenged males per group).

Since body weight of *dy*^*2J*^*/dy*^*2J*^ mice was significantly lower when compared to wild type mice (Fig 2A), we looked at the hallmarks of muscle atrophy. We measured the muscle fiber cross-sectional area in the gastrocnemius and triceps of 34 weeks old wild type and *dy*^*2J*^*/dy*^*2J*^ mice (Fig 4A). Gastrocnemius of *dy*^*2J*^*/dy*^*2J*^ mice consisted of a higher proportion of smaller fibers (*P*<0.05) with a myofiber area between 100–1000 μm^2^ (Fig 4B). The majority of myofibers in wild type mice had an area between 1500–2500 μm^2^. Furthermore, *dy*^*2J*^*/dy*^*2J*^ triceps also showed a shift towards smaller fiber sizes (*P*<0.05) when compared to wild type mice (Fig 4C). Namely, *dy*^*2J*^*/dy*^*2J*^ triceps contained a significantly higher number of fibers with an area between 100–500 μm^2^, whereas in wild type triceps the majority of fibers were between 500–2000 μm^2^. Comparing *dy*^*2J*^*/dy*^*2J*^ gastrocnemius to triceps muscle, triceps contained a higher proportion of >3000 μm^2^ than gastrocnemius indicating more atrophy in gastrocnemius induced by hind limb paralysis. No significant differences were found between genders.

To study atrophy further in *dy*^*2J*^*/dy*^*2J*^ skeletal muscles, we measured expression levels of genes involved in muscle atrophy such as *Gadd45a* and *Cdkn1a* (encoding p21^Cip1/Waf1^), *Csrp3* (encoding muscle LIM protein), *Ankrd1* and *Peg3* (encoding PW1/Peg3). This subset of genes is regulated via activating transcription factor-4 (*Atf4*) and their mRNA expression is highly induced after muscle starvation and denervation [15, 16]. Expression levels of the atrophic transcripts *Gadd45a*, *Cdkn1a*, *Csrp3* and *Ankrd1* were elevated in *dy*^*2J*^*/dy*^*2J*^ gastrocnemius compared to wild type, while *Peg3* levels were increased but did not reach significance (Fig 4D). Similar to the fiber size measurements, gastrocnemius had a more atrophic profile when compared to triceps (Fig 4D and 4E). In triceps, *Gadd45a* and *Csrp3* expression levels were significantly increased in *dy*^*2J*^*/dy*^*2J*^ mice, while the levels of *Cdkn1a*, *Ankrd1* and *Peg3* remained unchanged (Fig 4E). We also tested a set of genes involved in atrophy in the diaphragm but none of the atrophic markers differed between *dy*^*2J*^*/dy*^*2J*^ and wild type mice (Panel a in S1 Fig). Although the atrophic subset of genes mediated via *Atf4* was upregulated in *dy*^*2J*^*/dy*^*2J*^ gastrocnemius and triceps, the expression levels of muscle RING-finger protein-1 (*Murf-1/Trim63*) were comparable between *dy*^*2J*^*/dy*^*2J*^ and wild type gastrocnemius and diaphragm muscles (Panel b in S1 Fig). Levels of *Murf-1* were even significantly decreased in *dy*^*2J*^*/dy*^*2J*^, when compared to wild type triceps. These findings suggest that muscle atrophy in *dy*^*2J*^*/dy*^*2J*^ mice is activated via the *Atf4*-mediated pathway and not via the ubiquitin proteasome pathways [17, 18].

To exclude the possibility that the small fibers in gastrocnemius and triceps consisted of newly regenerated fibers, we measured gene expression of the myogenic markers *Myh3*, and *Myog*. Expression levels of *Myh3* were very low in *dy*^*2J*^*/dy*^*2J*^ and wild type muscles and did not differ between the genotypes. The expression levels of *Myog* were significantly increased in gastrocnemius muscles of *dy*^*2J*^*/dy*^*2J*^ mice, but comparable in triceps and diaphragm between genotypes. (Panel c in S1 Fig). This indicates that there is some ongoing regeneration in gastrocnemius but very limited to no regeneration activity in the other muscles.

#### Muscle fibrosis is elevated in *dy*^*2J*^*/dy*^*2J*^ mice

**Fig 5:**
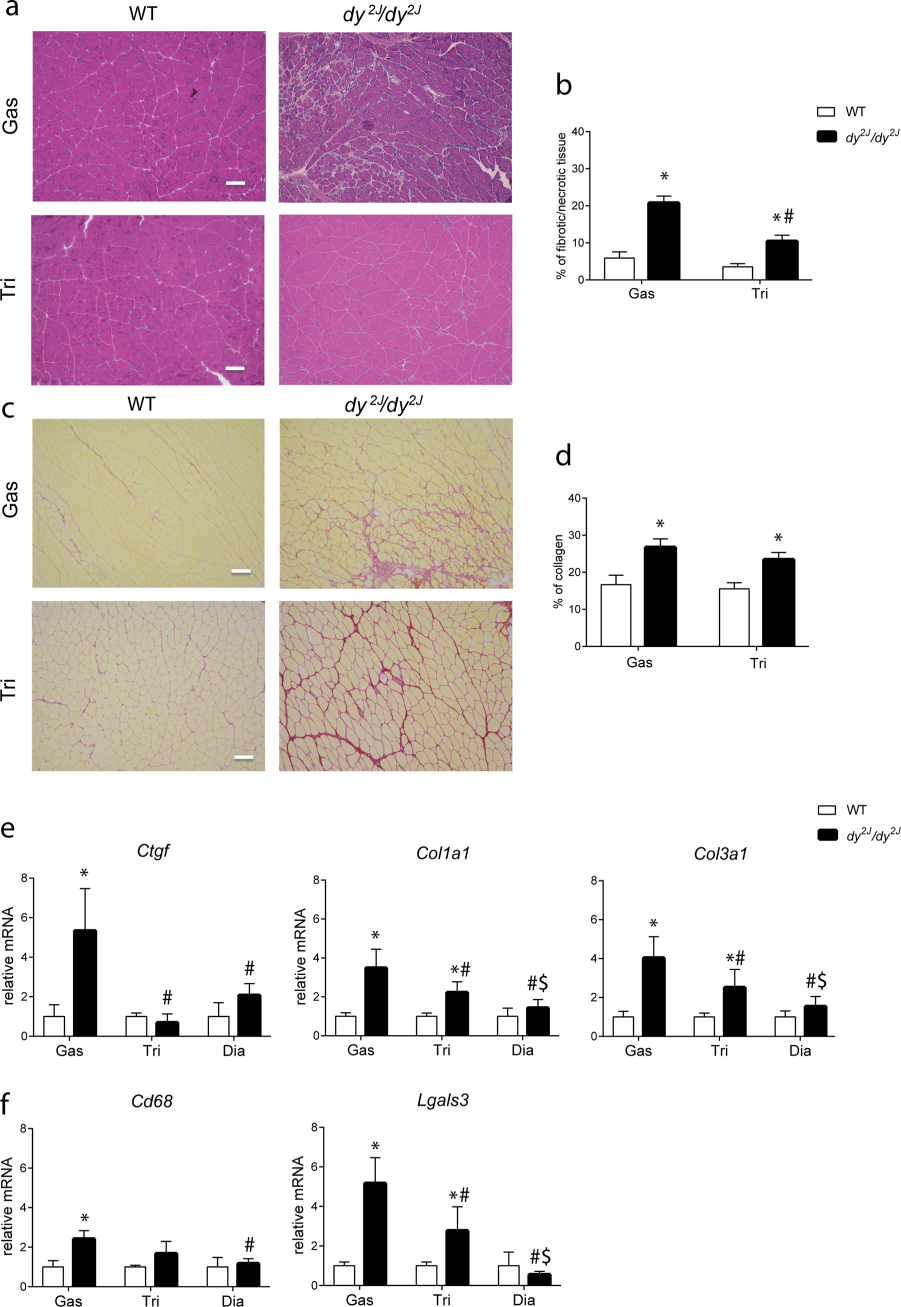
Increased muscle fibrosis and inflammation in *dy*^*2J*^*/dy*^*2J*^ mice. **(a)** Representative H&E-stained gastrocnemius and triceps from 34 weeks old male wild type and *dy*^*2J*^*/dy*^*2J*^ mice. Scale bar: 100 μm **(b)** Percentage of pathological area quantified on H&E stained sections. A significant increase (*P*<0.0001) in pathological tissue area was found in *dy*^*2J*^*/dy*^*2J*^ gastrocnemius and triceps relative to wild type muscles. Pathology was more pronounced (*P*<0.0001) in gastrocnemius than in triceps of *dy*^*2J*^*/dy*^*2J*^ mice. **(c)** Representative Sirius red-stained gastrocnemius and triceps from 34 weeks old male wild type and *dy*^*2J*^*/dy*^*2J*^ mice. Scale bar: 100 μm. (**d**) Percentage of collagen quantified on Sirius red stained sections. A significant increase in Sirius red positive area was found in *dy*^*2J*^*/dy*^*2J*^ gastrocnemius and triceps relative to wild type muscles. **(e)** Gene expression of fibrotic markers measured by qPCR. Gastrocnemius of *dy*^*2J*^*/dy*^*2J*^ mice expressed significantly higher levels of *Ctgf*, *Col1a1* and *Col3a1* (*P*<0.01) when compared to *dy*^*2J*^*/dy*^*2J*^ triceps, diaphragm and wild type muscles. *Col1a1*and *Col3a1* levels (*P*<0.05) were significantly higher in triceps muscle of *dy*^*2J*^*/dy*^*2J*^ mice than in wild type and *dy*^*2J*^*/dy*^*2J*^ diaphragm. **(f)** Gene expression of inflammatory markers measured by qPCR. A significant upregulation of *Cd68* transcript (*P*<0.01) was found in gastrocnemius of *dy*^*2J*^*/dy*^*2J*^ mice when compared to wild type mice and *dy*^*2J*^*/dy*^*2J*^ diaphragm. *Lgals3* levels were significantly higher (*P*<0.01) in *dy*^*2J*^*/dy*^*2J*^ gastrocnemius and triceps when compared to wild type and *dy*^*2J*^*/dy*^*2J*^ diaphragm. *Lgals3* levels of gastrocnemius exceeded (*P*<0.01) those of the triceps in *dy*^*2J*^*/dy*^*2J*^ mice. Data were normalized to *Gapdh*. Gas, gastrocnemius; Tri, triceps and Dia, diaphragm. * Indicates a significant difference from muscle type-matched wild type (WT) controls. # Indicates a significant difference from *dy*^*2J*^*/dy*^*2J*^ gastrocnemius muscle. $ Indicates a significant difference from *dy*^*2J*^*/dy*^*2J*^ triceps. Error bars represent ± SD, n = 5 (functionally challenged males per group). For Sirius red analysis: n = 5 functionally challenged wild type males and n = 4 functionally challenged *dy*^*2J*^*/dy*^*2J*^ males.

Haematoxylin and eosin (H&E) staining of the *dy*^*2J*^*/dy*^*2J*^ gastrocnemius revealed a high content of pathological tissue. Muscle pathology was less pronounced in the triceps and absent in wild type muscles (Fig 5A). Quantification of the H&E staining revealed a significant increase in the percentage of pathological tissue in both muscles of *dy*^*2J*^*/dy*^*2J*^ mice and the increase was significantly higher in *dy*^*2J*^*/dy*^*2J*^ gastrocnemius than in triceps (Fig 5B). To directly quantify collagen levels, Sirius red staining was used. Collagen content was significantly higher in gastrocnemius and triceps muscles of *dy*^*2J*^*/dy*^*2J*^ than in wild type. Levels were more pronounced in gastrocnemius than triceps of *dy*^*2J*^*/dy*^*2J*^ mice, but they did not reach significance (Fig 5C and 5D). To further validate this finding, we measured the relative gene expression of three different fibrosis-related genes: *Ctgf* [19], *Col1a1* and *Col3a1* [20] and two inflammation-related genes: *Cd68* and *Lgals3* [21]. Significantly higher levels of *Ctgf* were found in gastrocnemius of *dy*^*2J*^*/dy*^*2J*^ mice when compared to wild type muscles, *dy*^*2J*^*/dy*^*2J*^ triceps and diaphragm (Fig 5E). Comparable to histological data, levels of *Col1a1* and *Col3a1*were significantly increased in *dy*^*2J*^*/dy*^*2J*^ gastrocnemius and triceps when compared to wild type mice (Fig 5E). Furthermore, the gene expression of *Col1a1* and *Col3a1* significantly differed between muscle types of *dy*^*2J*^*/dy*^*2J*^ mice, where gastrocnemius expressed significantly higher levels when compared to triceps and diaphragm. In addition, *Col1a1* and *Col3a1* levels in triceps exceeded those of the diaphragm in *dy*^*2J*^*/dy*^*2J*^ mice. Levels of *Cd68* were increased in the *dy*^*2J*^*/dy*^*2J*^ gastrocnemius muscles compared to the wild type muscles and *dy*^*2J*^*/dy*^*2J*^ diaphragm (Fig 5F*)*. Levels of *Lgals3* were significantly increased in both gastrocnemius and triceps of *dy*^*2J*^*/dy*^*2J*^ mice relative to wild type mice, where *Lgals3* expression in gastrocnemius exceeded that of the triceps (Fig 5F).

To summarize, *dy*^*2J*^*/dy*^*2J*^ mice displayed the hallmarks of muscle atrophy such as a decrease in body weight, higher proportion of smaller atrophic myofibers and increased expression levels of genes associated with muscle starvation and denervation. As expected due to hind limb paralysis, the signs of muscle atrophy were most pronounced in gastrocnemius, which also showed higher levels of fibrosis and inflammation compared to the triceps and the diaphragm.

### Functional test regime does not interfere with the muscle pathology

**Fig 6:**
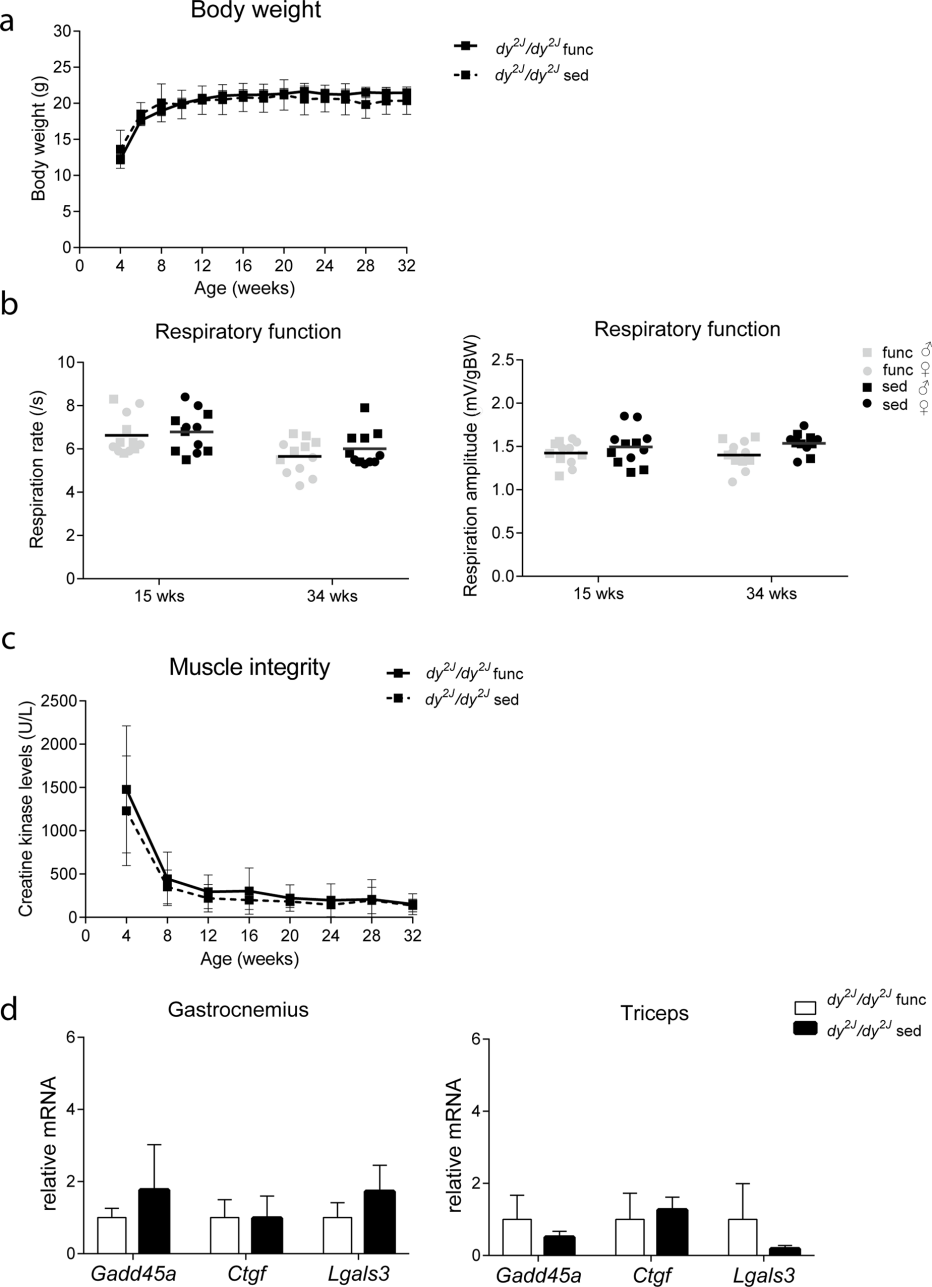
Functional test regime did not interfere with muscle pathology in *dy*^*2J*^*/dy*^*2J*^ mice. **(a)** Body weight did not differ between functionally challenged and sedentary mice. **(b)** Respiration rate and amplitude were comparable between functionally challenged and sedentary mice. **(c)** CK levels did not differ between functionally challenged and sedentary mice **(d)** Gene expression analysis of pathogenic markers measured by qPCR, normalized to *Gapdh* in gastrocnemius and triceps muscle of functionally challenged and sedentary mice. No differences were found in *Gadd45a*, *Ctgf* and *Lgals3* mRNA levels between functionally challenged and sedentary groups. Func, functionally challenged mice; sed, sedentary mice. Error bars represent in a and c ± SEM and in d ± SD. Sample sizes of body weight, respiratory function and CK analysis n = 6 males and 6 females group, while n = 5 males per group were used for gene expression analysis.

To study whether the functional test regime interfered with disease pathology in *dy*^*2J*^*/dy*^*2J*^ mice, sedentary groups were taken along. We assessed effects of exercise on body weight, respiratory function, plasma CK levels and expression of genes involved in muscle pathogenesis in *dy*^*2J*^*/dy*^*2J*^ mice. Body weight was comparable between functionally challenged and sedentary mice (Fig 6A). Respiration rate and amplitude did not differ between functionally challenged and sedentary mice (Fig 6B), neither did plasma CK levels (Fig 6C). Lastly, the expression of genes involved in muscle pathogenesis (*Gadd45a*, *Ctgf* and *Lgals3*) was not altered in functionally challenged mice compared to sedentary mice (Fig 6D). Therefore, we conclude that the functional test regime did not affect muscle pathology in *dy*^*2J*^*/dy*^*2J*^ mice.

## Discussion

Currently, there is no treatment available that directly corrects LAMA2 (merosin) deficiency in MDC1A patients or the associated neurological symptoms. However, recent studies in animal models and patients have advanced the understanding of underlying mechanisms of MDC1A [5]. Several mouse models have been developed for laminin-α2 chain-deficiency recapitulating the clinical MDC1A heterogeneity. However, the crucial elements for the development of new therapies, including comprehensive natural history data and standardized outcome measures are still largely lacking. This impedes pre-clinical research in MDC1A mouse models. Therefore, we collected natural history data and assessed applicability of outcome measures for laminin-α2 reduced *dy*^*2J*^*/dy*^*2J*^ mice.

There are several studies that provided some phenotypic data and assessed muscle function in *dy*^*2J*^*/dy*^*2J*^ mice. Although these studies gained insights on control *dy*^*2J*^*/dy*^*2J*^ mice, they were therapeutic intervention studies containing only few functional tests and up to four measured time points [22–24]. In contrast, our natural history study comprises a complete data set that provides a list of reliable and reproducible outcome measures for future pre-clinical studies. Moreover, this data set can be used to predict sample size for future therapeutic studies. In addition, the present study was done according to standardized operating procedures available from the TREAT-NMD Alliance. This makes it more reliable and useful for pre-clinical study designs. Additionally, several important aspects for pre-clinical study design were determined such as differences in disease pathology between genders, muscle types and whether functional test regime affects muscle pathology.

*Dy*^*2J*^*/dy*^*2J*^ mice were subjected to longitudinal, non-invasive muscle function assessments in a standardized manner, as previously described [12]. This allowed monitoring disease progression over time [25]. We identified that four limbs grip strength and the hanging tests were sensitive and reliable outcome measures to detect marked differences in performance between wild type and *dy*^*2J*^*/dy*^*2J*^ mice from the age of four weeks onwards. To be noted, the four limbs grip strength needs to be performed by one person, as the outcome of this test is highly variable between experimenters. Although the *dy*^*2J*^*/dy*^*2J*^ mice developed hind limb paralysis, the mice were still capable to complete those tests. In young non-paralyzed mice, the hanging tests assessed different capacities and functionalities due to difference in starting position [12]. Namely, in the two limb hanging test, mice needed to use their abdominal and pelvic muscles to bring their hind limbs and tail to the wire enabling them to use all four limbs during hanging. Here, fine movements of the hind paws are not needed, as crossing of the hind limbs over the wire is sufficient for hanging. Contrastingly, in the four limb hanging test, mice used fine motoric of all four paws from the start of the test onwards to hold onto the grid. Notably, in older mice with hind limb paralysis the distinction between these two tests disappeared, as both solely measured upper body strength and function. We also identified muscle functional tests that were less reliable or useful. Although the rotarod test revealed a significant difference between wild type and *dy*^*2J*^*/dy*^*2J*^ mice, this outcome measure showed high variability between individual wild type mice. Despite the large variation, there was still a clear difference between wild type and *dy*^*2J*^*/dy*^*2J*^ mice, owing to the fact that the *dy*^*2J*^*/dy*^*2J*^ mice perform very poorly. However, if future intervention studies improve functionality, the large variation may make it more difficult to pick up beneficial effects on muscle function with this test. The limitations of rotarod testing are similar to those previously discussed for *mdx* mice (model for Duchenne muscular dystrophy) [25]. We would not recommend this test for *mdx* and *dy*^*2J*^*/dy*^*2J*^ mice. Also the beam walk test, previously used to assess balance and coordination in mice [26, 27], was deemed unsuitable for untreated *dy*^*2J*^*/dy*^*2J*^ mice, as they were not able to traverse the beam due to hind limb paralysis. Only intervention studies in which a large therapeutic effect is expected could consider this test as outcome measure.

Unlike MDC1A patients who severely suffer from respiratory insufficiency, breathing function of *dy*^*2J*^*/dy*^*2J*^ mice was not very severely changed. Although, *dy*^*2J*^*/dy*^*2J*^ respiration rates were reduced due to the laminin-α2 deficiency [22], the mice were able to compensate by increasing their respiratory amplitude and thereby likely comply with their oxygen demand. To do so, functionally intact respiration muscles are required. This is in line with the mild pathology we observed in the diaphragm. CK is a measure for muscle fiber integrity and levels are elevated upon muscle damage. Plasma CK levels are widely used as a diagnostic marker in humans and as a therapeutic marker in pre-clinical animal models. In contrast to *e*.*g*. *mdx* mice with highly increased CK levels, the *dy*^*2J*^*/dy*^*2J*^ mice displayed nearly normal CK levels. Notably, the levels were slightly elevated in young (four to eight weeks of age) *dy*^*2J*^*/dy*^*2J*^ mice, which could still use their hind limbs. Daily use of these hind limb muscles likely resulted in decreased fiber integrity. With age, the mobility of the hind limbs decreased due to paralysis, corresponding to a normalization of their CK levels. We found that both genders fully manifest the disease phenotype showing no gender differences in muscle function. However, since genders might respond differently to therapies, we advise to evaluate whether this is the case before proceeding with using a single gender to minimize variation in pre-clinical studies.

Our histological and gene expression analyses revealed a specific pattern of muscle type involvement in *dy*^*2J*^*/dy*^*2J*^ mice, which needs to be taken into account for future pre-clinical studies. Namely, hind limb muscles were most severely affected showing atrophy, fibrosis and inflammation caused by immobilization and denervation [28]. Moreover, some dystrophic characteristics were found in forelimb muscles but these were less pronounced. As previously mentioned, in contrast to the models of other muscular dystrophies, which display very severe pathology in diaphragm [29] of *dy*^*2J*^*/dy*^*2J*^ mice was found to be barely affected. One key characteristic of the *dy*^*2J*^*/dy*^*2J*^ model is muscle atrophy. We here demonstrated that ATF4 but not MURF-1 mediated pathways are associated with atrophy and muscle damage in this mouse model. Moreover, there are much more pathways associated with atrophy and muscle damage that have been previously described for mouse models of MDC1A [9]. Besides muscle degradation pathways, autophagy–lysosome pathway [30] and apoptosis [31] have been shown to be over-activated and thereby contribute to muscle damage and dysfunction in *dy*^*2J*^*/dy*^*2J*^ mice and other mouse models for MDC1A. This study was longitudinal and we assessed muscle pathology at 34 weeks of age. Future studies should include cross-sectional analyses. These will allow a direct comparison between muscle function and pathology at younger age, when mice are still capable to use their hind limbs. Furthermore, the cross-sectional studies will provide better insights about muscle atrophy and damage in relation to the disease progression and age. Moreover, additional studies to assess muscle function and muscle damage in *dy*^*2J*^*/dy*^*2J*^ mice below four weeks of age would be useful. These studies may provide novel markers or outcome measures that can be used to assess the efficacy of therapeutic approaches.

## Conclusion

The present study offers a natural history dataset for *dy*^*2J*^*/dy*^*2J*^ mice, based on which we make a number of recommendations. These recommendations can be useful in setting up an experiment for the evaluation of new therapeutic targets.

1. Functional outcome measures: functional *in vivo* tests such as grip strength test, hanging tests, and whole-body plethysmography can be very useful. In contrast, we do not advise to use rotarod, due to high variability in wild type mice. The beam walk test should only be used when a huge therapeutic effect is expected, since untreated *dy*^*2J*^*/dy*^*2J*^ mice could not comply with this test due to hind limb weakness.
2. Terminal outcome measures: histological analyses such as H&E and Sirius red stainings, fiber size analysis and gene expression analyses of atrophic, fibrotic and inflammatory markers are useful outcome measures.
3. Gender selection: although we did not find any differences between genders in muscle function or pathology, we recommend using a single gender for pre-clinical trials once it has been established that both genders respond to a therapy equally.
4. Muscle selection: we found a specific pattern of muscle involvement and advise to include several muscles in a pre-clinical study evaluation, e.g. gastrocnemius (severely affected muscle) and triceps (moderately affected).
5. Sample size: We here used six mice per group for functional *in vivo* tests and five mice per group for terminal outcomes such as histological and gene expression analyses. This sample size allowed us to pick up the differences between wild type and affected mice. Sample sizes for therapeutic interventions will depend on the expected effect size. Our dataset could be helpful to determine future sample size using dedicate sample size formula [32, 33].
6. Age of treatment: muscle impairment is detectable already at four weeks of age. We recommend starting a treatment as soon as possible as the hind limb paralysis deteriorates over time.

## Supporting information

S1 Checklist ARRIVE (Animal Research: Reporting of *In Vivo* Experiments) guidelines checklist that is required for reporting of research using animals. (DOCX)

S1 Fig Gene expression of atrophic and regenerative markers measured by qPCR. **(a)** Subset of atrophic genes in diaphragm did not differ between *dy*^*2J*^*/dy*^*2J*^ and wild type mice **(b)** *Murf-1* levels were similar between *dy*^*2J*^*/dy*^*2J*^ and wild type gastrocnemius and diaphragm muscles and significantly decreased in *dy*^*2J*^*/dy*^*2J*^ triceps. **(c)** Expression levels of *Myog* were comparable between *dy*^*2J*^*/dy*^*2J*^ and wild type triceps and diaphragm, but significantly increased in *dy*^*2J*^*/dy*^*2J*^ gastrocnemius compared to wild type. Data were normalized to *Gapdh*. Gas, gastrocnemius; Tri, triceps and Dia, diaphragm. * Indicates a significant difference from muscle type-matched WT controls Error bars represent ± SD, n = 5 functionally challenged males per group.
(TIF)

S1 Table Primer sequences used for qPCR analysis. (PDF)

## Abbreviations

CMD: Congenital muscular dystrophy
MDC1A: Merosin deficient congenital muscular dystrophy 1A
LAMA2: Laminin Subunit Alpha 2
CK: Creatine kinase
dy^2J^/dy^2J^: mouse model of laminin α2 (merosin) deficient
WT: Wild type mice
