# Supplementary material for: Natural disease history of the dy2J mouse model of laminin α2 (merosin)-deficient congenital muscular dystrophy
Source: PLoS One. 2018 May 15;13(5):e0197388. doi: 10.1371/journal.pone.0197388 (PMC5953480; doi:10.1371/journal.pone.0197388)

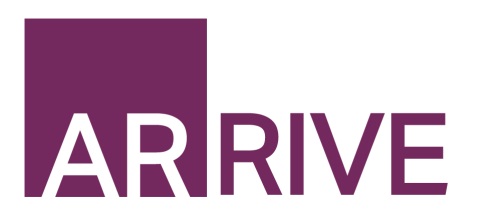


The ARRIVE Guidelines Checklist

Animal Research: Reporting In Vivo Experiments

S. Pasteuning-Vuhman^1^, K. Putker^1^, C.L. Tanganyika-de Winter^1^, J.W. Boertje-van der Meulen^1^, L. van Vliet^1^, M. Overzier^1^, J.J. Plomp^2^, A. Aartsma-Rus^1^, M. van Putten^1^

^1^ Department of Human Genetics Leiden University Medical Centre, Leiden, The Netherlands ^2^ Department of Neurology Leiden University Medical Centre, Leiden, The Netherlands.

|  | | ITEM | RECOMMENDATION | Section/ Paragraph |
| --- | --- | --- | --- | --- |
| 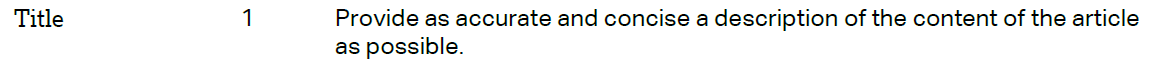 | | | Title |  |
| 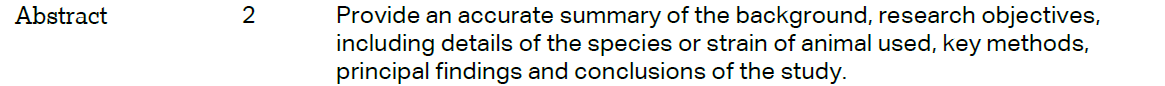 | | | Abstract |  |
| INTRODUCTION | | |  |  |
| 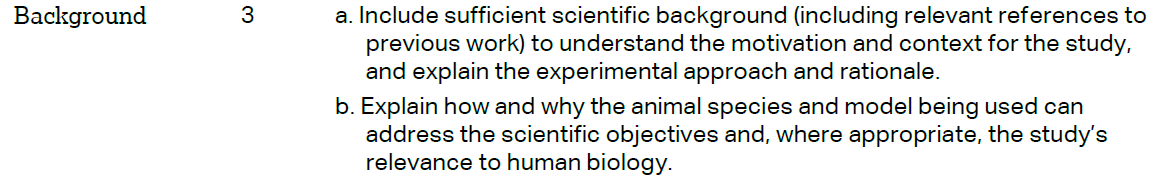 | | | Page 3  Page 4 |  |
| 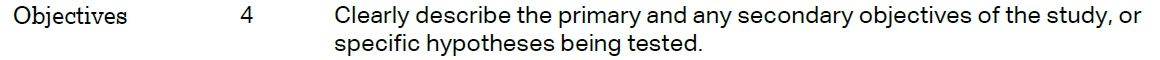 | | | Page 4 |  |
| METHODS | | |  |  |
| 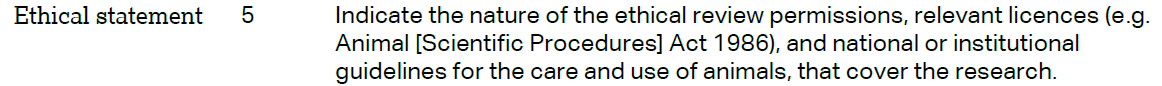 | | | Page 4-5 |  |
| 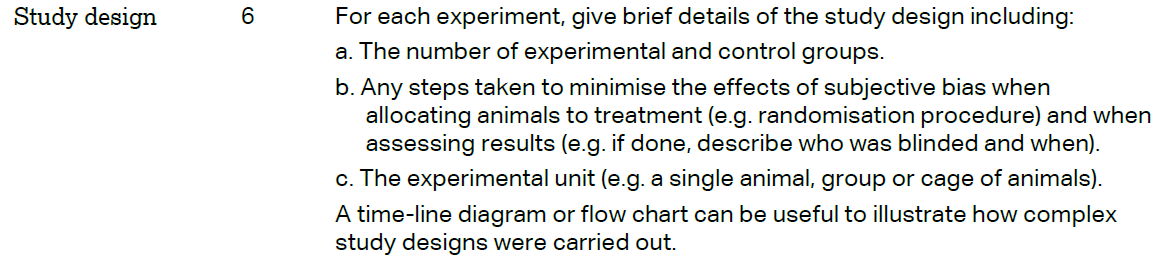 | | | Page 5  Page 5  Page 5  Figure 1 |  |
| 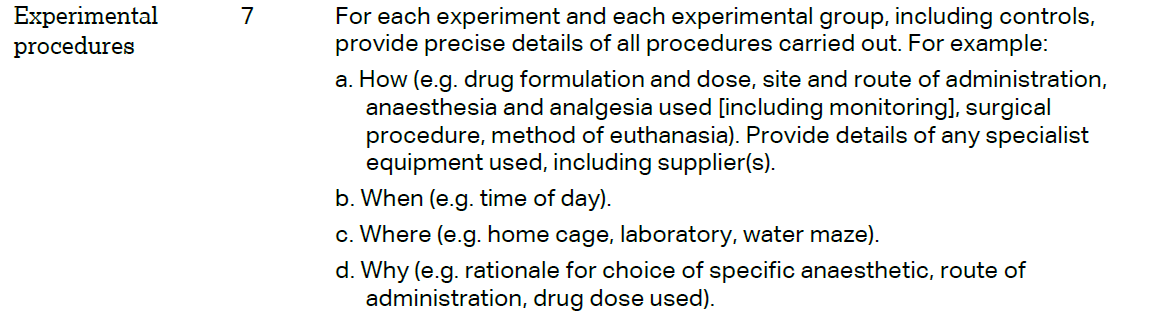 | | | Page 5-9 |  |
| 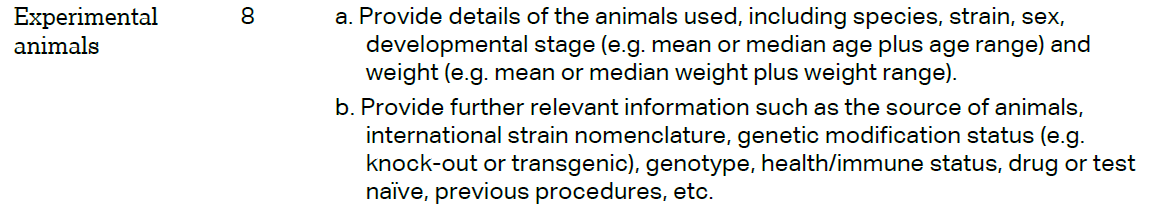 | | | Page 4-5  Page 4-5 |  |

The ARRIVE guidelines. Originally published in *PLoS Biology*, June 2010^1^

| 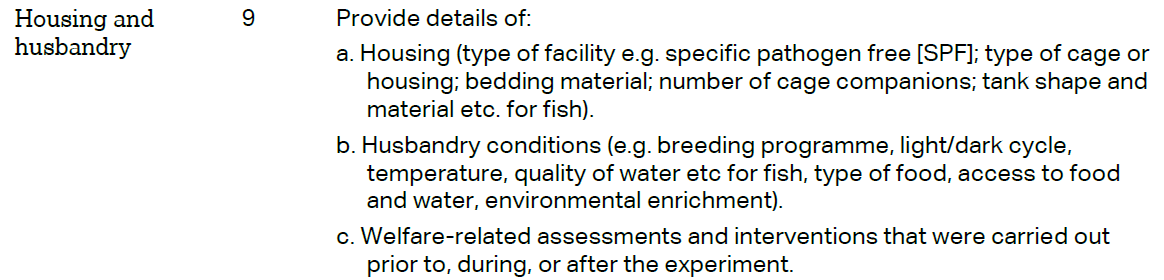 | Page 4-5  Page 4-5  Page 5-9 | |
| --- | --- | --- |
| 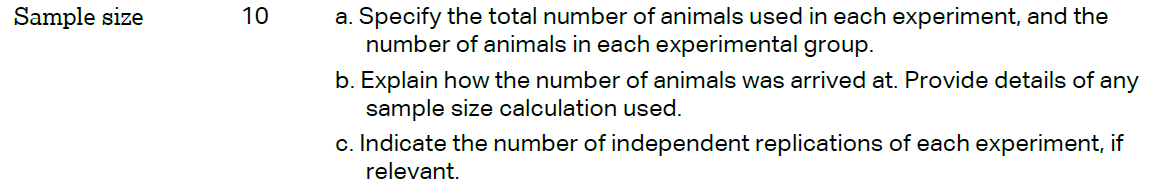 | Page 5 | |
| 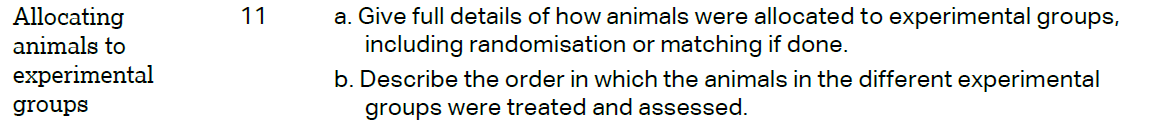 | Page 4-5 | |
| 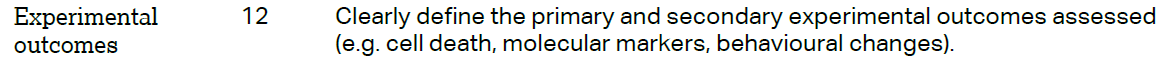 | Page 5-9 | |
| 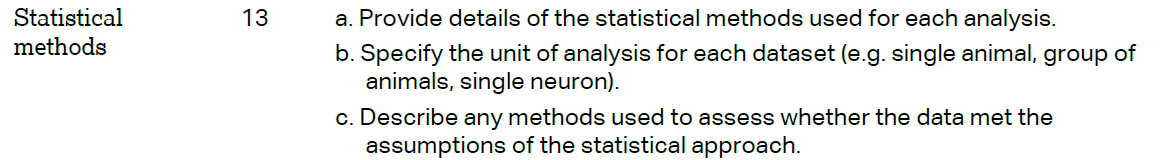 | Page 9-10 | |
| RESULTS |  | |
| 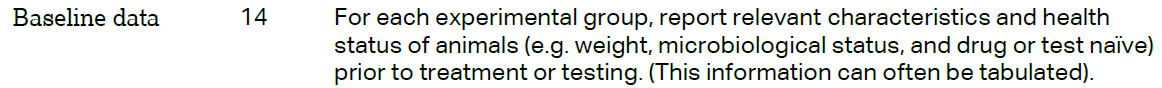 | Figure 2 and 3 | |
| 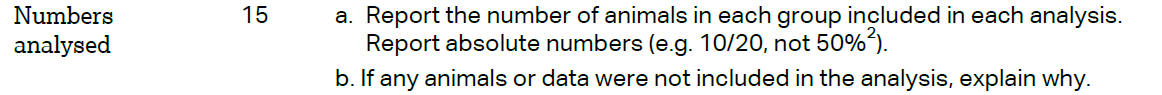 | See Figure legends | |
| 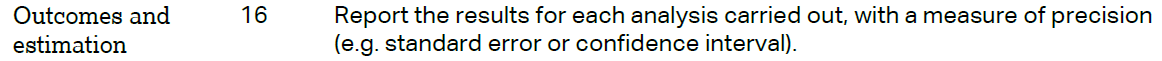 | Figures 2-6 | |
| 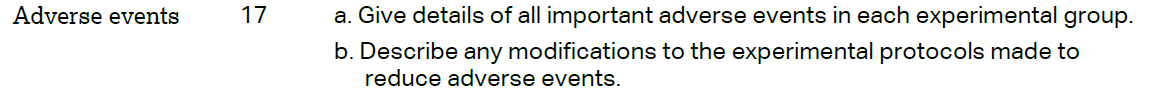 | No adverse events were observed | |
| DISCUSSION |  | |
| 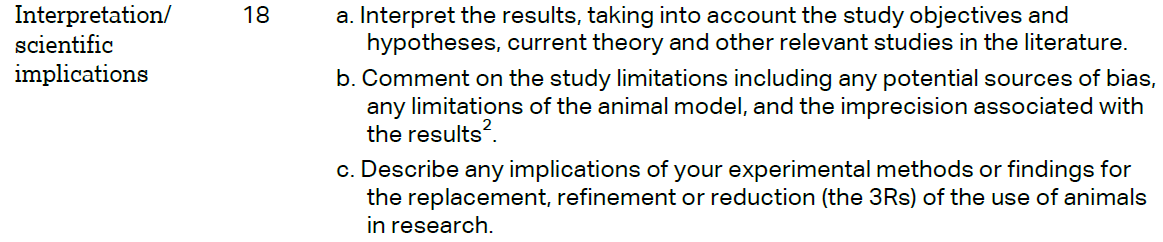 | Discussion  Page 18,19, 20  Conclusion | |
| 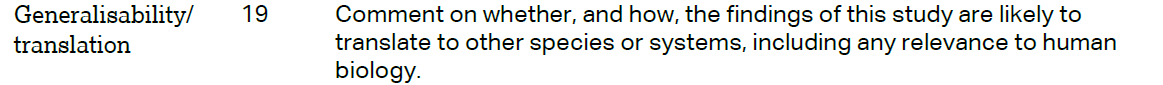 | Conclusion  (These data useful for future preclinical studies) | |
| 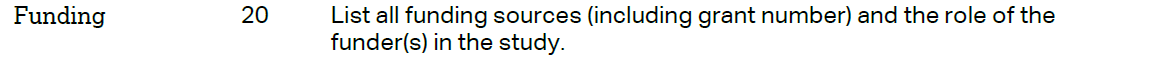 | | This work was supported by grants from ZonMw (Project 113302001),NeurOmics (FP7, Agreement 2012-305121) and AFM (grant number 20251). |


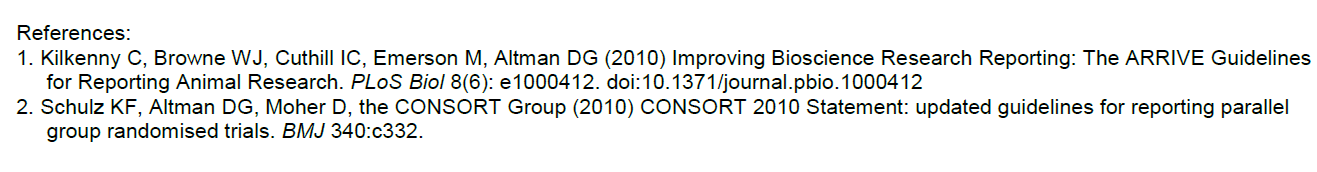

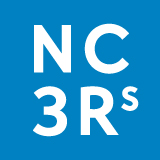

Supplement: S1 Checklist — (DOCX) [file pone.0197388.s001.docx]
