# Supplementary figures and images for: Natural disease history of the dy2J mouse model of laminin α2 (merosin)-deficient congenital muscular dystrophy
Source: PLoS One. 2018 May 15;13(5):e0197388. doi: 10.1371/journal.pone.0197388 (PMC5953480; doi:10.1371/journal.pone.0197388)

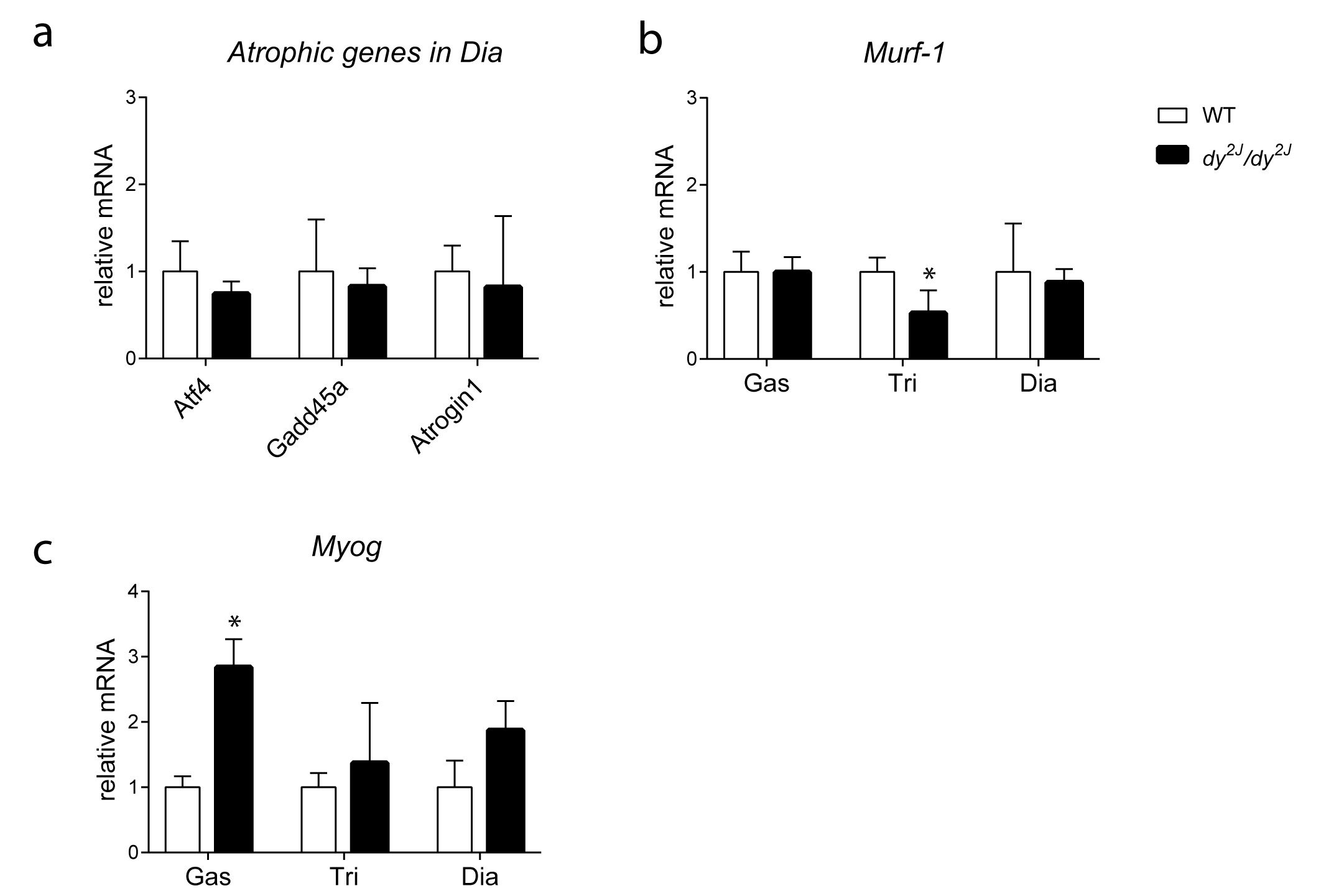

Supplement: S1 Fig — (a) Subset of atrophic genes in diaphragm did not differ between dy2J/dy2J and wild type mice (b) Murf-1 levels were similar between dy2J/dy2J and wild type gastrocnemius and diaphragm muscles and significantly decreased in dy2J/dy2J triceps. (c) Expression levels of Myog were comparable between dy2J/dy2J and wild type triceps and diaphragm, but significantly increased in dy2J/dy2J gastrocnemius compared to wild type. Data were normalized to Gapdh. Gas, gastrocnemius; Tri, triceps and Dia, diaphragm. * Indicates a significant difference from muscle type-matched WT controls Error bars represent ± SD, n = 5 functionally challenged males per group. (TIF) [file pone.0197388.s002.tif]
