# Supplementary material for: Natural disease history of the dy2J mouse model of laminin α2 (merosin)-deficient congenital muscular dystrophy
Source: PLoS One. 2018 May 15;13(5):e0197388. doi: 10.1371/journal.pone.0197388 (PMC5953480; doi:10.1371/journal.pone.0197388)
Supplement: S1 Table — (PDF) [file pone.0197388.s003.pdf]

Gene	Full gene name	Primer sequence (5’–3’)	Direction
Gadd45a	Growth arrest and DNA-damage inducible alpha	GCTGCCAAGCTGGTCAAC	forward
		TCGTCGTCTTCGTCAGCA	reverse
Cdkn1a	Cyclin-dependent kinase inhibitor 1a	CTAGGGGAATTGGAGTCAGG	forward
		AGACAACGGCACACTTTGCT	reverse
Csrp3	Cysteine and glycine rich protein 3	TGAAAAGACGGTCTACCATGC	forward
		GTGCTGTCCAGAGCTTTCCT	reverse
Ankrd1	Ankyrin repeat domain 1	GCGATCGTGGAGAAGTTAATG	forward
		GACATCTGCGTTTCCTCCAC	reverse
Peg3	Paternally-expressed gene 3 protein	AGGGGCAGAAGCAGAGATGT	forward
		GGAAGAGAAAGATCCCGTTG	reverse
Atf4	Activating transcription factor 4	GGAATGGCCGGCTATGG	forward
		TCCCGGAAAAGGCATCCT	reverse
Atrogin-1	Atrogin-1	AGAGTCGGCAAGTCTGTGCT	forward
		TCAGCCTCTGCATGATGTTC	reverse
Murf-1/Trim63	Muscle RING finger protein-1	TGTGCAAGGAACACGAAGAC	forward
		CCAGCATGGAGATGCAGTTA	reverse
Myh3	Embryonic myosin heavy chain	CGCAGAATCGCAAGTCAATA	forward
		CAGGAGGTCTTGCTCACTCC	reverse
Myog	Myogenin	CCCAACCCAGGAGATCATTT	forward
		GTCTGGGAAGGCAACAGACA	reverse
Ctgf	Connective tissue growth factor	AGCTGGGAGAACTGTGTACG	forward
		GCCAAATGTGTCTTCCAGTC	reverse
Col1a1	Collagen type I alpha 1	ATGTTCAGCTTTGTGGACCT	forward
		CAGCTGACTTCAGGGATGT	reverse
Col3a1	Collagen type III alpha 1	TCCCCTGGAATCTGTGAATC	forward
		TGAGTCGAATTGGGGAGAAT	reverse
Cd68	Cluster of differentiation 68	CCAATTCAGGGTGGAAGAAA	forward
		GAGAGAGACAGGTGGGGATG	reverse
Lgals3	Lectin, galactoside-binding, soluble, 3	CAACCATCGGATGAAGAACC	forward
		TTCCCACTCCTAAGGCACAC	reverse
Gapdh	Glyceraldehyde-3-Phosphate Dehydrogenase	TCCATGACAACTTTGGCATTG	forward
		TCACGCCACAGCTTTCCA	reverse
